# Detection of quorum sensing virulence factor genes and its consanguinity to antibiotic sensitivity profile in the clinical isolates of *Pseudomonas aeruginosa*

**DOI:** 10.22038/IJBMS.2023.67981.14992

**Published:** 2023

**Authors:** Vadla Shravani, Girija Aseervatham Selvi Selvi, Himabindu Mantravadi

**Affiliations:** 1 Department of Microbiology, School of Allied Health Sciences, Mallareddy University, Hyderabad, Telangana, India; 2 Department of Microbiology, Saveetha Dental College and Hospitals, Saveetha Institute of Medical and Technical Sciences (SIMATS), Chennai, India; 3 Department of Microbiology, Mallareddy Institute of Medical Sciences, Hyderabad, Telangana, India

**Keywords:** Multidrug-resistant strains, Pseudomonas aeruginosa, Quorum sensing activity, Resistant strains, Virulence

## Abstract

**Objective(s):**

Virulent strains of *Pseudomonas aeruginosa* exhibit multidrug resistance by intrinsic and extrinsic mechanisms which are regulated by quorum sensing signalling systems. This includes the production of auto-inducers and their transcriptional activators to activate various virulence factors resulting in host infections. The present study is thus aimed to detect the virulence factor production, quorum sensing activity, and susceptibility pattern of *P. aeruginosa* to antibiotics from clinical specimens.

**Materials and Methods:**

A total of 122 isolates of *P. aeruginosa* were phenotypically characterized by standard protocols and were categorized into MDR and non-MDR based on the antibiotic susceptibility profiles. Pyocyanin, alkaline protease and elastase production were assessed by qualitative and quantitative methods. Crystal violet assay was carried out for the quantification of biofilms. The genetic determinants of virulence were detected by PCR.

**Results:**

Out of the 122 isolates, 80.3% of isolates were MDR and the production of virulence factors was in positive correlation with the presence of genetic determinants and 19.6% were non-MDR, but still showed the production of virulence factors, as confirmed by both phenotypic and genotypic methods. Few carbapenem-resistant strains were detected which did not show the production of virulence factors by both methods.

**Conclusion:**

The study concludes, though the strains were non-MDR, they were still capable of producing the virulence factors which may be responsible for the dissemination and chronicity of the infection caused by* P. aeruginosa*.

## Introduction

Bacteria communicate through a well-developed system termed quorum sensing (QS) (1, 2). It is a cell-density-dependent mechanism through which bacteria co-ordinate different activities including the production of various virulence factors (3, 4). Intercellular signalling is accomplished through small molecules termed auto-inducers. *Pseudomonas aeruginosa* possesses at least two well-defined, interrelated QS systems, *las, rhl*, that control the production of biofilm formation and different virulence factors like elastases, alkaline protease, pyocyanin, etc., (3, 4). Each QS system consists of two components, the autoinducer synthases which are *lasI* and *rhlI*, and their cognate transcriptional auto regulators *lasR* and *rhlR*, respectively (3, 4). The *lasI* controls *rhlR* operon and activates the transcription of *lasB* and *las* A. *rhlI* is up-regulated by the *rhlI* system and positively regulates *lasB* and pyocyanin production. The production of these virulence factors disrupts the host’s normal microbiota leading to an increase in bacterial pathogenicity and its survival resulting in high morbidity and mortality (5). The bacilli being multidrug-resistant becoming more difficult to eradicate by evading the immune response of the host causes pathological damage, which is crucial for the pathogenesis and further establishment of infection (6, 7). *P. aeruginosa* is the commonly isolated species from chronic wound infection and is considered to be a potent biofilm producer. Biofilms produced by these organisms act as a barrier in wound healing and exhibits higher rates of antimicrobial therapy resistance (8, 9). *Las* and *rhl* systems are not only involved in biofilm formation but also regulate various gene expressions necessary for the production of virulence factors (10*)*. Extensive studies document the positive correlation and association between the biofilm-producing ability, the production of various virulence factors, and multidrug resistance phenotypic expression in *P. aeruginosa* (11). However, the association of the virulence and resistant determinants varies with different clinical strains observed using various methods. Given this background, the present study is aimed to demonstrate the relationship between the expression of some important virulence determinants, biofilm-forming ability, and multidrug-resistant phenotypes among the clinical strains of *P. aeruginosa*. 

## Materials and Methods

***Bacterial strains***

A total of 122 non-duplicate isolates were characterized from the clinical samples received from the outpatient unit, inpatient wards and ICUs of Mallareddy Medical College and Teaching Hospital, Hyderabad, Telangana, between the periods of August 2017 to December 2019. The clinical specimens include sputum *(*n=33*)*, swabs from wounds *(*n=19*)*, urine *(*n=13*)*, endotracheal aspirates *(*n=22*)*, tracheobronchial fluids *(*n=21*)*, blood *(*n=9*)*, and specimens from soft tissues *(*n=12*)*. All the samples were collected following the aseptic techniques, ruling out colonizing flora, and were immediately processed in the microbiology laboratory. 

***Identification and growth conditions***

The isolates were processed and identified according to standard operating procedures(12) and maintained at –20°C in glycerol stock for further process. Frozen stocks were subcultured periodically onto nutrient agar slants and kept at 4°C. Fresh cultures were retrieved periodically by transferring a loop full of growth from the slants to the nutrient broth and cultured overnight at 37°C, for the assays.

***Antibiotic susceptibility testing***

Susceptibility testing against routine antibiotics for the *P. aeruginosa* isolates was performed by the Kirby-Bauer disc diffusion method according to Clinical and Laboratory Standards Institute (CLSI) guidelines(12) with antibiotics, ceftazidime (30 µg), gentamicin (10 µg), tobramycin (µg), piperacillin-tazobactam (30/6 µg), amikacin (30 µg), aztreonam (30 µg), cefepime (30 µg), ciprofloxacin (5 µg), imipenem (10 µg), meropenem (10 µg), doripenem (10 µg), and levofloxacin (5 µg).

***Assessment of virulence factors***

*Qualitative assessment of pyocyanin, elastase, and protease*

Pyocyanin, elastase, and alkaline protease were demonstrated by inoculating *P. aeruginosa* isolates on *Pseudomonas* P agar (peptone 20 g/l, magnesium chloride 1.40 g/l, potassium sulfate 10 g/l, and agar- agar 15 g/l, pH 7.2 ± 0.2, (Hi-media, Mumbai, India), Elastin nutrient agar (15) and Milk agar plates (18) respectively. This is followed by 48 hr of incubation at 37 °C, whereas P agar plates require longer incubation, up to 4-5 days (13). Production of blue colonies (14), elastinolysis (16), and milk hydrolysis as clear transparent zones surrounding the colonies- are considered indicative of pyocyanin, elastase, and alkaline protease respectively.

*Biofilm*

Biofilm assay is performed using 96 well microtiter plate method, according to O’Toole G.A (22). Briefly, overnight broth culture of P. aeruginosa was prepared in 1:100 dilutions with fresh medium. 100 µl of the dilution is dispensed into each well of a microtiter plate, incubated at 37 °C for 4-24 hr. One hundred twenty-five μl of 0.1% crystal violet is added to the wells for staining of the biofilms, followed by 10-15 min of incubation at room temperature. Planktonic cells and media components are removed by thoroughly submerging the plate in water 3-4 times. The plate was blotted vigorously on the stack of paper and then dried overnight for a few hours by placing them upside down. The wells can be photographed for qualitative assay.

***Quantitative assessment***

*Pyocyanin assay*

Pyocyanin extraction was done by adding 3 ml of chloroform with 5ml of culture supernatant with diffused pigment after centrifugation of broth culture to remove the cells. Pyocyanin was then re-extracted by adding 1ml of acidified water (0.2 M HCl) to the culture supernatant which gives a pink to a red solution. The absorbance of pyocyanin was measured at 520 nm for quantification using a microtiter plate reader (19)

*Elastase assay*

Quantification of elastase was performed following the method of Gupta *et al.* (20). Briefly, 200 µl of the culture supernatant was added to 200 µl of 100 mM Tris-HCl buffer (pH 7.0) supplemented with 1 mM CaCl_2_. To this, 1mg of elastin-congo red is added in tubes, and kept at 37 °C for 2 hr, on a rotator. After 2 hr, the preparation is mixed with 300 µl of 0.7 M sodium phosphate buffer (pH 6.0) and centrifuged for 5 mins at 1500 rpm at 4° C. The preparation is then transferred to microtiter plates for reading absorbance 495_nm_ filters with a microtiter plate reader, and the results are expressed in units per milliliter (U/ml).

*Protease assay*

*Pseudomonas* isolates, which were positive for the screening test with milk agar showing a zone of milk hydrolysis were further considered for the protease assay (21). Quantification of protease was done by adding 1% azo-casein in 0.05 M tris HCl with 400 µl of culture supernatant, incubated in a water bath at 37 °C for 1 hr. One-hundred thirty-five µl of 35% trichloroacetic acid (TCA) is added. The mixture is kept on ice for 15 min to stop the reaction. The mixture was centrifuged for 10 min at 13000 rpm. 750 µl of the centrifuged mixture is taken and added to 750 µl of 1 N NaOH. The preparation was then transferred into the microtiter wells and absorbance was read at 440_nm_ filters with a microtiter plate reader. Blank is prepared by adding 400 µl of culture supernatant first with 135 µl of 35% TCA and then with 1% azo casein in 0.05N Tris HCl, with further incubation in a water bath at 37 °C for 1 hr. The mixture is kept on ice for 15 min followed by centrifugation for 10 mins. The final absorbance is measured at 440 nm after 750 µl of the preparation is added with an equal volume of 1N NaOH.

*Biofilm assay*

For quantification, 125 μl of 30% acetic acid in water is added to each microtiter plate with biofilm, to solubilize the dye and incubated at RT for 10-15 min. In order to read the absorbance at 550 nm on a microtiter plate reader, 125 μl of solubilized CV is then transferred to a new flat-bottomed microtiter dish (22).

***QS Virulence factors gene detection by PCR***

Extraction of bacterial DNA was done by PureFast**® **Bacterial/fungal DNA mini spin commercial purification kit. The total reaction mixture for PCR was 25 μl, with 10 μl of purified DNA, 10 μl of master mix, and 5 μl of the primer mix. Oligonucleotide primers obtained from HELINI Biomolecules, Chennai, India, for *lasl, lasR, rhll, rhlR, lasB, aprA, phzM,* and *phzS*, were used and were tabulated (Table 1). PCR programming was done with an initial denaturation at 95 °C for 5 min, followed by denaturation, annealing, and extension at 94 °C for 30 sec, 58 °C for 30 sec and 72 °C for 30 sec for 35 cycles. The final extension is at 72 °C for 5 min. Gene detection was done on 2% agarose gel, added with 5 μl of ethidium bromide dye. The band pattern was viewed by running electrophoresis at 50V under a transilluminator.

***Statistical analysis***

A Pearson product-moment correlation was run to determine the relationship between MDR and non-MDR positive and negative strains for various virulence factor variables. All the data were analysed on SPSS software, version 25. Relevant tables and graphs were done using Microsoft Excel.

## Results

Out of 122 isolates of *P. aeruginosa*, 80.3% (n=98) were MDR & 19.6% (n=24) were non-MDR, susceptible to many antibiotics (Table 4). Among the MDR strains, 81.9% (n=100) of the strains were resistant to gentamycin, tobramycin, ciprofloxacin, and levoflocacin, followed by amikacin 89.3% (n=109), ceftazidime 80.3% (n=98), piperacillin 79.5% (n=97), cefepime 74.5% (n= 91), while the lowest resistance was noticed in Imepenem 7.3% (n=9), dorepenem 9% (n=11) and Meropenem 11.4% (n=14). (Table 2).

**Figure 1:**
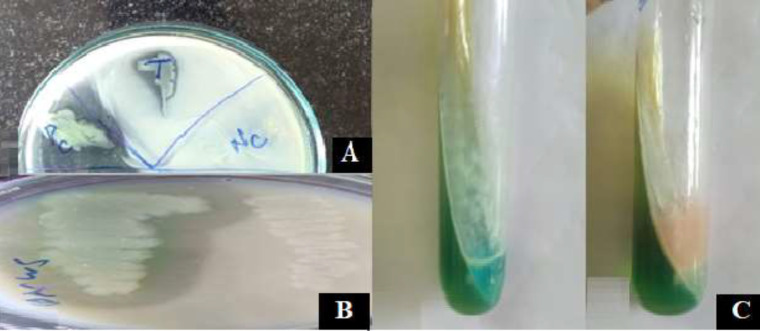
Detection of virulence factors produced by the clinical strains of *Pseudomonas aeruginosa *A. Detection and quantification of biofilm formation by crystal violet staining method, B & C. Qualitative and quantitative assessment of pyocyanin, D. Qualitative assessment of elastase & E. Alkaline protease production

**Figure 2:**
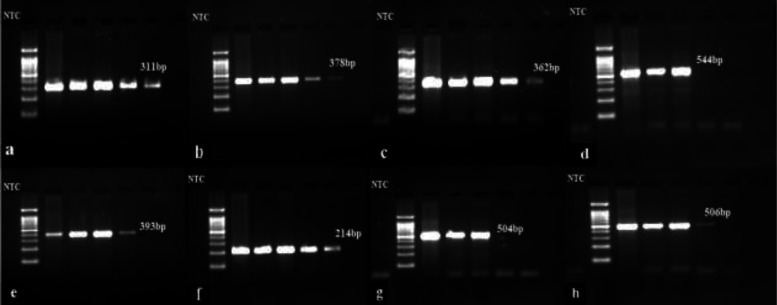
Electropherogram of the virulent gene determinants with lane 1 showing NTC (Negative template control), lane 2 showing the DNA ladder (1500bp) and lane 3,4,5,6 showing the positive amplicons of a. *lasI* (311 bp), b. *lasr* (378 bp), c. *rhll* (362 bp), d. *rhlr* (544 bp), e. *phzM* (393 bp), f. *phzS* (214 bp), g. *lasB* (504 bp), h. *aprA* (506 bp) and lane 8 with negative strains

All MDR strains, for all QS virulence genes like *lasl* (98/100%), *lasR* (98/100%), *rhll* (98/100%), *rhlR *(94/95%) showed approximately 100% positivity; in non-MDR strains 19 for *lasl & rhll*, 17 for *lasR*, 11 for *rhll* were positive and 5 for *lasl*, 7 for *lasR*, 5 for *rhll* and 9 for *rhlR* were negative for the same. The same positive percentages were identified for all virulence factors ranging from *phzM* (98/100%), *phzS* (96/97.9%) for pyocyanin; *lasB* (96/97.9%) for elastase & *aprA* (98/100%) isolates were positive in MDR strains whereas 19 (79.1%) and 22 (91.6%) isolates were negative for pyocyanin, elastase and 24 (100%) were positive for *aprA* for protease among non-MDR strains (Figure 1). The isolates that were multidrug resistant were generally expressing all quorum sensing genes. In our study, the majority of the non-MDR isolates were also identified with genes for virulence factors by PCR methods (Tables 3, 4) (Figure 2).

**Table 1:** Primer sequences for detection of virulence factor genes in *Pseudomonas aeruginosa*

**Target gene**	**Gene primer**	**PCR Product**
*Lasl*	F: GGCGCGAAGAGTTCGATAAA R: TCCAGAGTTGATGGCGAAAC	311bp
*lasR*	F: GATCCTGTTCGGCCTGTT R: TGCAGTGCGTAGTCCTTG	378bp
*Rhll*	F: GCTCTCTGAATCGCTGGAAG R: GCAGGCTGGACCAGAATATC	362bp
*RhlR*	F: GGATGTTCTTGTGGTGGAAGT R: GGCTTCGATTACTACGCCTATG	544bp
*phzM*	F: CTGCTGCGCGTAATTTGATAC R: GCTTCAGGTAGCTGTAGAAGTC	393bp
*phzS*	F: GGAAAGCAGCAGCGAGATAC R: ATGGATCGAGTACTGCGGATAG	214bp
*lasB*	F: GACACCAGCGGATAGAACAT R: CAACGTCTCCTACCTGATTCC	504bp
*aprR*	F: GTGCTGACCCTGTCCTATTC R: GTGTTCTGCTCTTCCCAGTAG	506bp

**Table 2:** Distribution of antibiotic susceptibility pattern in clinical isolates of *Pseudomonas aeruginosa* (n=122)

**Name of antibiotic **	**No. of Resistant isolates**	**No. of Sensitive isolates**
Ceftazidime	98 (80.3%)	24 (19.6%)
Cefepime	91 (74.5%)	31 (25.4%)
Gentamycin	100 (81.9%)	22 (18%)
Tobramycin	100 (81.9%)	22 (18%)
Amikacin	109 (89.3)	13 (10.6%)
Aztreonam	91 (74.5%)	31 (25.4%)
Ciprofloxacin	100 (81.9%)	22 (18%)
Levofloxacin	100 (81.9%)	22 (18%)
Piperacillin Tazobactum	97 (79.5%)	25 (20.4%)
Meropenem	14 (11.4%)	108 (88.5%)
Dorepenem	11 (9%)	112 (91.8%)
Imepenem	9 (7.3%)	118 (96.7%)

**Table 3:** Results of phenotypic methods by qualitative and quantitative methods of virulence factors in MDR isolates of* Pseudomonas aeruginosa* (n= 98)

**Virulence factors**	**Qualitative method**		**Quantitative method**	
	**Positive**	**Negative**	**Positive**	**Negative**
Biofilm production	98 (100%)	0	98 (100%)	0
Pyocyanin	86 (87.8%)	12 (12.2%)	81 (82.6%)	17 (17.3%)
Elastase	71 (72.4%)	27 (27.5%)	68 (69.38%)	30 (30.6%)
Protease	72 (73.4%)	26 (26.5%)	70 (71.4%)	28 (28.5%)

**Table 4:** Quorum sensing gene expression among MDR and non-MDR strains of *Pseudomonas aeruginosa *by PCR (n= 122)

	**MDR strains of ** ***Pseudomonas aeruginosa*** ***n= 98*** ** (80.3%)**		**Non-MDR strains of ** ***Pseudomonas aeruginosa*** ***n = 24*** ** (19.6%)**	
**Quorum sensing biofilm genes**				
	**Positive**	**Negative**	**Positive**	**Negative**
*las l*	98 (100%)	0	19 (79.1%)	5 (20.8%)
*las r*	98 (100%)	0	17 (70.8%)	7 (29.1%)
*rhl l*	98 (100%)	0	19 (79.1%)	5 (20.8%)
*rhl r*	94 (95.9%)	4 (4.0%)	11 (45.8%)	9 (37.5%)
**Pyocyanin**				
*phzM*	98 (100%)	0	19 (79.1%)	5 (20.8%)
*phzS*	96 (97.9%)	2 (2.04%)	22 (91.6%)	2 (8.3%)
**Elastase**				
*las b*	96 (97.9%)	2 (2.04%)	22 (91.6%)	2 (8.3%)
**Protease**				
*apr A*	98 (100%)	0	24 (100%)	0

## Discussion

Based on the reports of the “Burden of antimicrobial resistance cooperative group”, over 700,000 infections, 4.95 million deaths, and ~900,000 disability-adjusted life years (DALY) were owing to MDR microorganisms (23). Non-fermenting gram-negative microorganisms extensively contribute to the overall communicable disease burden, especially in hospitalized individuals currently associated with the increasing trend of morbidity and mortality. Nearly 20-60% of hospital-acquired infections (HAI’s) are caused by drug-resistant strains (24, 25). This is evident in the World Health Organization’s (WHO) published report, in which carbapenem-resistant *P. aeruginosa* (CR-PA), carbapenem-resistant, and/or ESBL-producing *Enterobacterales*, *Acinetobacter* sp., were all identified as “critical priority” pathogens among the drug-resistant pathogens causing nosocomial infections (26). *P. aeruginosa* remains one of the foremost pathogens in the healthcare setting causing recalcitrant infections (27). Many multifactorial virulent determinants play an important role in its pathogenesis and further establishment of the disease. 

Overexpression of the efflux pump, and less permeability of the outer membrane, are innate resistance mechanisms, whereas acquiring resistance or mutation in genes encoding for porins, efflux pumps, and chromosomal beta-lactamase are included in acquired resistance mechanisms of *P. aeruginosa*. With all these known mechanisms, the organism acquires the ability to cause infection by manipulating the host-pathogen interaction (28). Amidst many virulent determinants in *P. aeruginosa* infections, biofilm production is considered an important factor behind pathogenicity. The formation of biofilms facilitates the chronicity of infections and reduces the effectiveness of antimicrobial treatment. The co-existence of virulence factors contributed by the quorum-sensing system and multidrug resistance makes *P. aeruginosa* a highly potent organism of interest in most research studies. An attempt was thus made to screen for the drug-resistant strains of *P. aeruginosa* prevalent in our health care setting. The present study documents an average of 81% of the strains resistant to aminoglycosides, and quinolones followed by cephalosporin, considering the group as MDR & 19.6% of non-MDR were observed. Whereas, Gajdács *et al.* (29), reported 44% of quinolone and cephalosporin resistance in MDR strains and 55.96% in non-MDR strains. In our study, 81.9% of resistance against ciprofloxacin was recorded which correlates with the study conducted by Algun *et al.* (30) and Mohanasoundaram (31).

Among the cephalosporins, resistance against ceftazidime and cefepime was observed in 80% and 74% of the strains respectively, correlating with the results observed by Ankita and Raja (32). The increased prevalence of ceftazidime resistance in *P. aeruginosa* might be related to modifications in PBP2 (33) and chromosomally encoded ampicillin C-type (AmpC) beta-lactamases, which are normally expressed at a low level, being induced by administration of many antibiotics leads to high-level resistance to cephalosporins (34, 35). Additionally, selective pressure for survival in the presence of antimicrobial agents is a major determinant for the emergence of resistant strains (36). Resistance to aminoglycoside seems to be higher in our study ranging up to 97%, which is in accordance with the study conducted by Abidi* et al. *(37). Among several mechanisms of resistance reported, it has been evidenced that up-regulation of the efflux system MexXY-OprM and the presence of aminoglycoside modifying enzymes (38, 39) like aminotransferases to a lesser extent are mainly encoded for the aminoglycoside resistance mechanism. Drug inactivation by plasmid or chromosome-encoded enzymes harbored by resistant strains typically results in such resistance. Although enzyme-independent resistance results from defects in uptake and accumulation which is termed dubbed impermeability resistance is also at common place. (40)

Evaluation of the virulent determinants among the MDR and the non-MDR strains rendered some interesting findings in the present study. Biofilm formation assayed by microtiter plate methods showed 98 isolates as biofilm formers in MDR isolates of *P. aeruginosa* and 19 non-MDR strains were also detected with genes for biofilms by PCR. The distribution of isolates with a difference in biofilm-forming capacities failed to show pronounced variations among the MDR non-MDR categories in our study, which was in line with the hypothesis of Cepas *et al.* (41), Singhai *et al.* (42), Karami *et al.* (43).

Based on our data, no significant differences were observed in biofilm and virulence factor gene detection between MDR and non-MDR strains of *P. aeruginosa*, where it was detected with the same genes in non-MDR strains similar to MDR strains. This analysis supports the data given by Yamani *et al.* (44), and Lima *et al.* (45), and is inconsistent with the findings of Choy *et al.* (46) & Jablameli *et al.* (47). The capacity of *P. aeruginosa* to form a varying degree of biofilm formation may additionally portray the variations exhibited among the species to establish infections in humans (48). Quorum sensing is the vital mechanism responsible for the regulation and expression of virulence factors and biofilm formation capacities and their related genes. *P. aeruginosa* has three special interconnected quorum sensing systems namely *las*, *rhl* and *pqs* which facilitate the organism’s survival in unfavourable conditions by identifying the signalling molecules in its surroundings (49). However, several antimicrobials even have the flexibility to affect the quorum sensing system in *Pseudomonas *sp., either by directly engaging the target gene or by degrading the signal molecules (50). 

Among 122 wild clinical strains, 98 (100%) MDR isolates showed the presence of *lasl, lasR, rhll* and 94 (95.9%) were expressing *rhlR*. In 24 non-MDR isolates, 19 (79.1%) were positive for *lasl, rhll*, 17 (70.8%) for *lasR* and 11(45.8%) for *rhlR*. The above percentage of isolates are in line with work done by Elnegery *et al.* (51) who reported 96% of isolates among 50 total strains positive for QS *lasR* and *rhl*R biofilms gene production. And also proved 99% of isolates were extremely QS-proficient strains with producing QS virulence factors. According to the report of La Sarre B, the production and regulation of QS virulence factors like protease and elastase are under the control of the *las* system, pyocyanin and biofilm is regulated by *rhl* system (52*)*. Pyocyanin production which is under the control of *rhl* system, has proven to have the ability to inhibit the electron transport chain, have vast antimicrobial activity, and is known to cause cell damage in *P. aeruginosa *(53*). *Our study reported the detection of *phzS* and *phzM* genes encoding for pyocyanin were up-regulated and identified by PCR in 98% of MDR and 23% of non-MDR isolates, these findings are in contrast to the study conducted by Khadim and Marjani who proved only 35% of *P. aeruginosa* isolates producing pyocyanin in burn patients. 

Protease production by the pathogen is highly significant in the pathogenesis of infection which degrades host tissue and aids the organism in growth and invasiveness. Protease enzyme coded by *aprA* gene controlled by the *rhlR* system (50) In our study all 122 (100%) isolates including MDR and non-MDR are proven to have protease the same genes, the percentage is in concordance with Elnegery *et al.* (51) and Khalil *et al.* (54) who reported 85% and 95% of protease production among patients with burn wounds. 

Detection of *lasR* was found in less virulent strains (56) irrespective of the above statement, our study resulted in 100% detection of the gene marker for *lasR* in all MDR and non-MDR isolates. The same results when compared to other studies conducted by El-Khasaab *et al.* (56) with 93%, and MF Mahmoud *et al.* (57) with 77.8%, were in concordance with *lasR* genes detected by PCR in patients isolated with *P. aeruginosa* in wound infections. *lasR* system is known to regulate the production of elastase coded by *lasB* gene which is a potent T2SS proteolytic enzyme have a varied substrate like connective tissue of elastin, collagen, fibronectin, and laminen (58). Comparing our results of detection of *lasB* for elastase with the work done by other researchers (59-61), who encrypted 100% positivity, prove it as an important factor for survival of *P. aeruginosa* in various clinical scenarios. 

## Conclusion

We conclude our findings by stating that there was a correlation between QS, biofilm formation and virulence factors and their specific genes in MDR *P. aeruginosa*, isolated from the clinical strains. The same virulence determinants were also observed in the non-MDR strains, leading to complications in nosocomial patients. It will send a clear-cut message to clinicians concerning the alarming state of resistance in *P. aeruginosa*, as well as how even non-MDR strains are also detected with virulence genes. The study thus strongly emphasizes the periodical monitoring of these virulent and resistant determinants among both MDR and non-MDR strains of *P. aeruginosa *during antimicrobial treatment in order to combat the menace of nosocomial infections. 

## Authors’ Contributions

V S wrote and drafted the manuscript. S GAS conceived, designed, supervised, and reviewed the manuscript. H M reviewed and proof read the final manuscript.

## Conflicts of Interest

The authors declare that there is no conflict of interest.
